# A case of neuroendocrine carcinoma in the hepatic hilar lymph nodes concomitant with an adenocarcinoma of the gallbladder

**DOI:** 10.1186/s12957-016-1039-6

**Published:** 2016-11-14

**Authors:** Haruka Okada, Yoichiro Uchida, Naomi Matsuzaki, Toru Goto, Satoshi Nishimura, Akira Kurita, Takafumi Nishimura, Shujiro Yazumi, Hiroaki Terajima

**Affiliations:** 1 Department of Gastroenterological Surgery and Oncology, The Tazuke Kofukai Medical Research Institute, Kitano Hospital, 2-4-20 Ohgimachi, kita-ku, Osaka City, Osaka 530-8480 Japan; 2 Department of Pathology, The Tazuke Kofukai Medical Research Institute, Kitano Hospital, Osaka, Japan; 3 Department of Gastroenterology and Hepatology, The Tazuke Kofukai Medical Research Institute, Kitano Hospital, Osaka, Japan; 4 Department of Medical Oncology, The Tazuke Kofukai Medical Research Institute, Kitano Hospital, Osaka, Japan; 5 Present Address: Department of Surgery, National Hospital Organization Kyoto Medical Center, 1-1, Fukakusamukaihatacho, Fushimi-ku, Kyoto City, Kyoto 612-8555 Japan

**Keywords:** Neuroendocrine carcinoma, Neuroendocrine tumor, Gallbladder cancer, Intestinal metaplasia

## Abstract

**Background:**

Neuroendocrine tumors (NETs) are rare especially in the gallbladder. They have not been elucidated in the pathogenesis, clinicopathological characteristics, and treatment options.

**Case presentation:**

We present a 76-year-old woman with a gallbladder tumor and hepatic hilar lymph node swelling. The lymph node biopsy demonstrated neuroendocrine carcinoma (NEC). We performed cholecystectomy, hepatic hilar lymphadenectomy, extrahepatic biliary duct resection, and hepaticojejunostomy prior to chemotherapy. Pathological examination revealed the gallbladder mass was an adenocarcinoma invading to the muscular layer without any NEC components, whereas the hepatic hilar lymph nodes were filled with high-grade NEC cells with negligible area of adenocarcinoma. The patient received general chemotherapy consisting of carboplatin and etoposide, but a recurrence in the para-aortic lymph nodes occurred 4 months after surgery.

**Conclusions:**

We report a rare case of NEC of the hepatic hilar lymph nodes that were concomitant with an adenocarcinoma of the gallbladder. High-grade NEC generally has an aggressive behavior and an optimal treatment strategy should be chosen for each patient.

## Background

Neuroendocrine tumors (NETs) are a heterogeneous group of tumors arising from diverse sites, with the majority occurring in the gastrointestinal tract and the bronchopulmonary system. Gallbladder NETs are particularly rare, accounting for only 0.34% of all NETs and less than 1% of all tumor types arising in the gallbladder [1, 2]. In the 2010 World Health Organization (WHO) classification of tumors of the digestive tract, NET is classified into NET G1, NET G2, and neuroendocrine carcinoma (NEC) based on mitotic count and Ki-67 index [3]. Here, we report a rare case of NEC in the hepatic hilar lymph nodes concomitant with a gallbladder adenocarcinoma.

## Case presentation

**Fig. 1:**
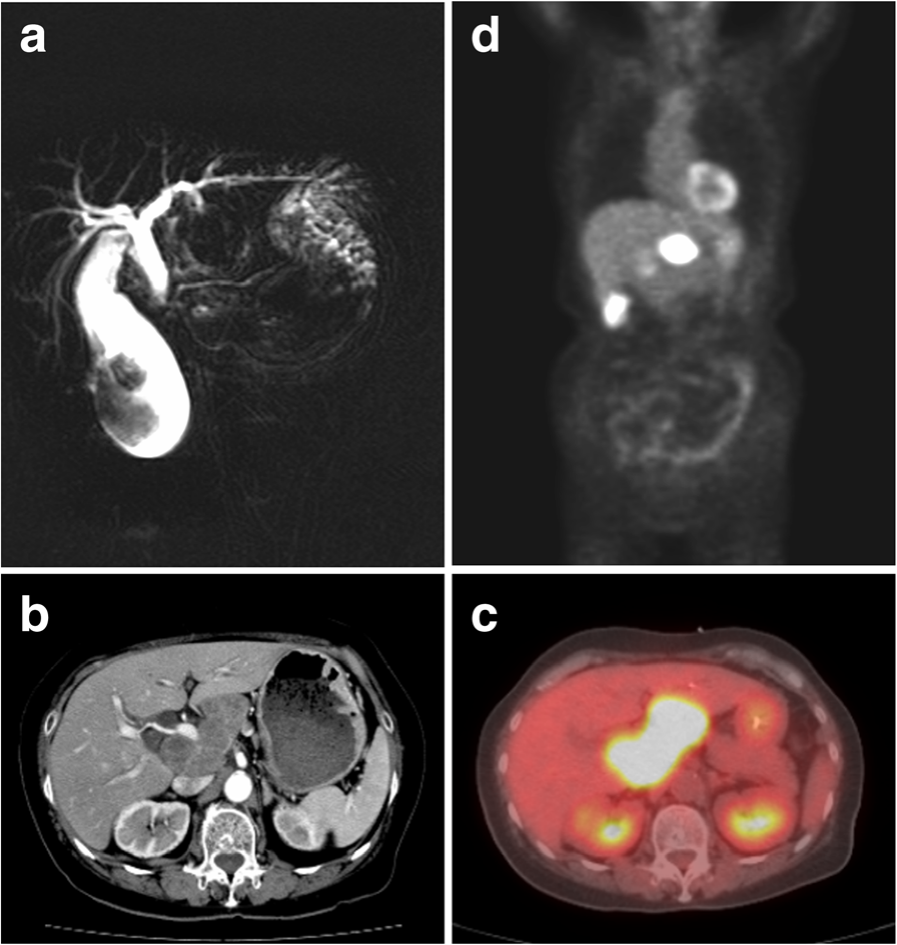
Preoperative imaging studies. Magnetic resonance cholangiopancreatography (MRCP) (**a**) and contrast-enhanced abdominal computed tomography (CT) scan (**b**) show a mass in a gallbladder with bulky hepatic lymph nodes surrounding the hepatic hilum. **c**, **d** Positron emission tomography/computed tomography (PET/CT) shows abnormal FDG uptake in the gallbladder (SUVmax 7.8) and the lymph node (SUVmax 13.4). No other metastases were identified

A 76-year-old woman presented to our hospital with complaints of epigastralgia since a day prior to admission. Laboratory data on admission revealed an elevation of aminotransferase, alanine aminotransferase, ɤ-guanosine triphosphate, and alkaline phosphatase. Serum total bilirubin and tumor markers, carcinoembryonic antigen (CEA), carbohydrate antigen 19-9 (CA19-9), SPan-1, and neuron-specific enolase (NSE), were all within normal ranges. Abdominal computed tomography (CT) and magnetic resonance cholangiopancreatography (MRCP) showed a mass in an enlarged gallbladder and bulky hepatic lymph nodes surrounding the hepatic hilum (Fig. 1a, b). There were also no apparent lesions in upper and lower gastrointestinal endoscopy. Endoscopic ultrasound-guided fine-needle aspiration (EUS-FNA) was performed to obtain tissue from the hilar lymph node. Immunohistochemical staining of the specimen identified diffuse positivity for keratin, CD56, and synaptophysin in the tumor cells, which is consistent with NEC. An endoscopic naso-gallbladder drainage (ENGBD) catheter was placed, and the bile cytology revealed class V malignant cells. Therefore, positron emission tomography/computed tomography (PET/CT) examination was performed to evaluate other primary or metastatic lesions. It revealed that no other accumulated lesions were identified, and the accumulation of ^18^F fluorodeoxyglucose (FDG) was in the gallbladder (SUVmax 7.8) and lymph nodes (SUVmax 13.4) (Fig. 1c, d). On the basis of these findings, the most likely diagnosis was a gallbladder NEC that was confined to the regional hepatic hilar lymph nodes metastasis.

Finally, we decided to perform surgical resection prior to chemotherapy because of concerns about complications developing from mechanical obstruction of the hepatic hilum by the enlarged lymph node. She underwent cholecystectomy, hepatic hilar lymphadenectomy, extrahepatic biliary duct resection, and hepaticojejunostomy. The bulky lymph nodes were totally resected as “en bloc”. There were no apparent residual lesions surgically. The postoperative course was uneventful and she was discharged on the tenth day after surgery.

**Fig. 2:**
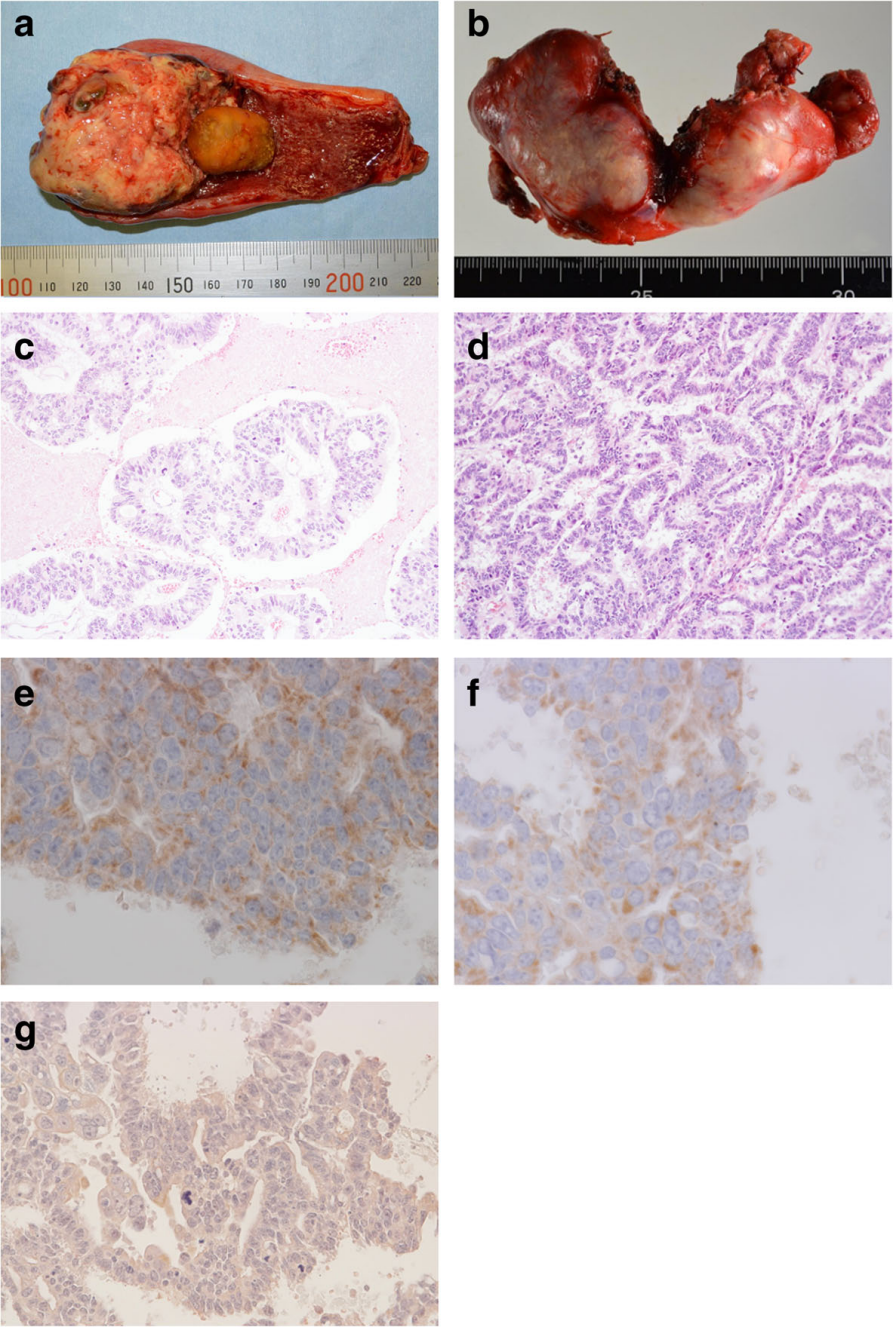
Macro and microscopic findings of the gallbladder. Gallbladder tumor with stone (**a**) and hepatic hilar lymph nodes (**b**). The tumor consists of well to moderately differentiated tubular adenocarcinoma (hematoxylin and eosin (H&E) stain, **c** ×200 and **d** ×400). The tumor cells in the gallbladder are slightly positive for **e** synaptophysin (×400) and **f** CD56 (×400), but negative for **g** chromogranin A (×400)

Macroscopically, the tumor was 58 × 42 mm in size and was located in the fundus, which contained a yellowish gallstone (Fig. 2a). A portion of the hepatic hilar lymph nodes (71 × 37 mm) was also excised separately (Fig. 2b). Microscopic examination of the gallbladder revealed a moderate to well differentiated tubular adenocarcinoma infiltrating from the mucosa to the muscular layer, but not the serosal surface, without any NEC components (Fig. 2c, d). The tumor cells in the gallbladder are slightly positive for synaptophysin and CD56, but negative for chromogranin A (Fig. 2e–g). The resection margin from the liver bed was negative for tumor cells. The epithelium around the carcinoma showed intestinal metaplasia with the goblet cells (Fig. 4a), which area was stained by alcian blue (Fig. 4b).

**Fig. 3:**
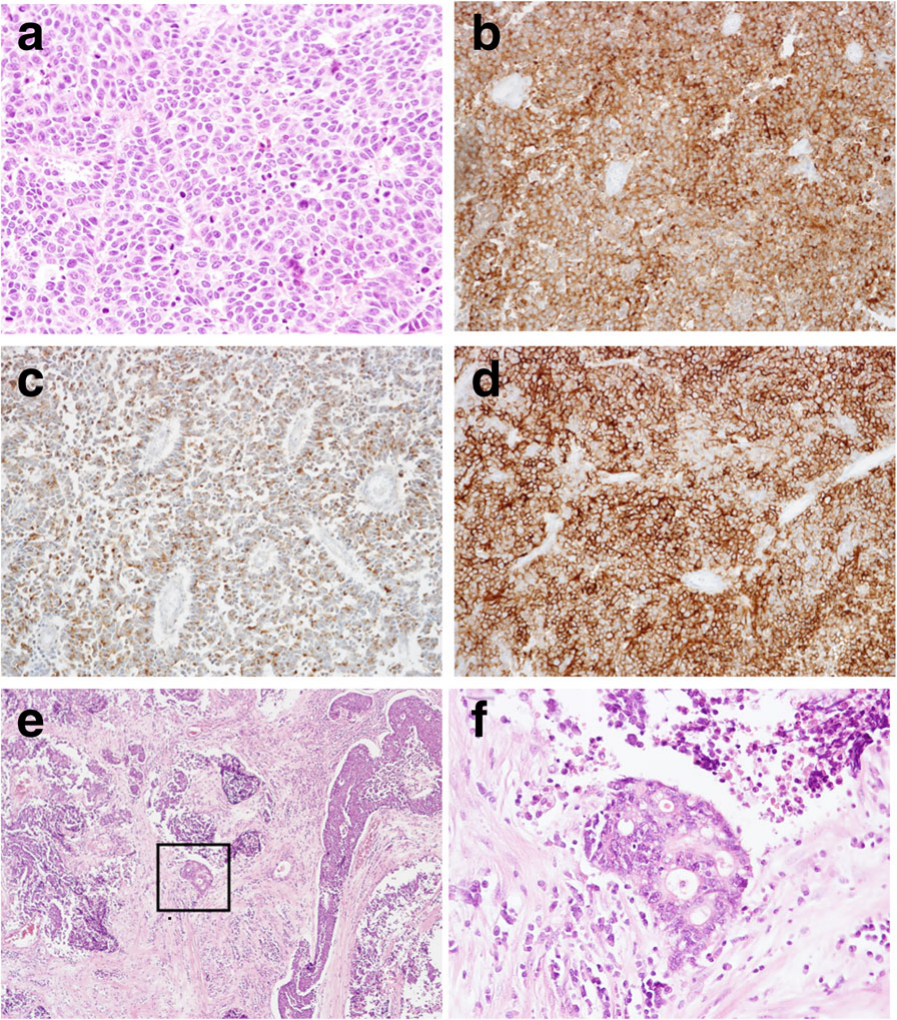
Microscopic findings of a hepatic hilar lymph node. **a** A sheet of differentiated small to large neuroendocrine carcinoma cells fills up the lymph node (hematoxylin and eosin (H&E) stain, ×400). The tumor cells in the lymph nodes are positive for **b** synaptophysin (×400), **c** chromogranin A (×400), and **d** CD56 (×400). The component of adenocarcinoma H&E stain **e** ×40, **f** ×200

On the other hand, the hepatic hilar lymph nodes were composed of small round tumor cells with hyperchromatic nuclei and scant cytoplasm (Fig. 3a). Some of the tumor cells were large and had vesicular nuclei. The tumor cells were arranged in sheets, cords, or in a trabecular or rosette fashion and were interspersed with focal necrosis. They were immunohistochemically positive for CD56, synaptophysin, and chromogranin A (Fig. 3b–3d). The mitotic count was 24 per 10 high-power microscopic fields, and the Ki-67 proliferation index was 70–80%, consistent with NEC. The surgical dissection margin of the hepatic lymph nodes was microscopically cauterized within the tumor cells. There was no invasion to the extrahepatic biliary duct.

**Fig. 4:**
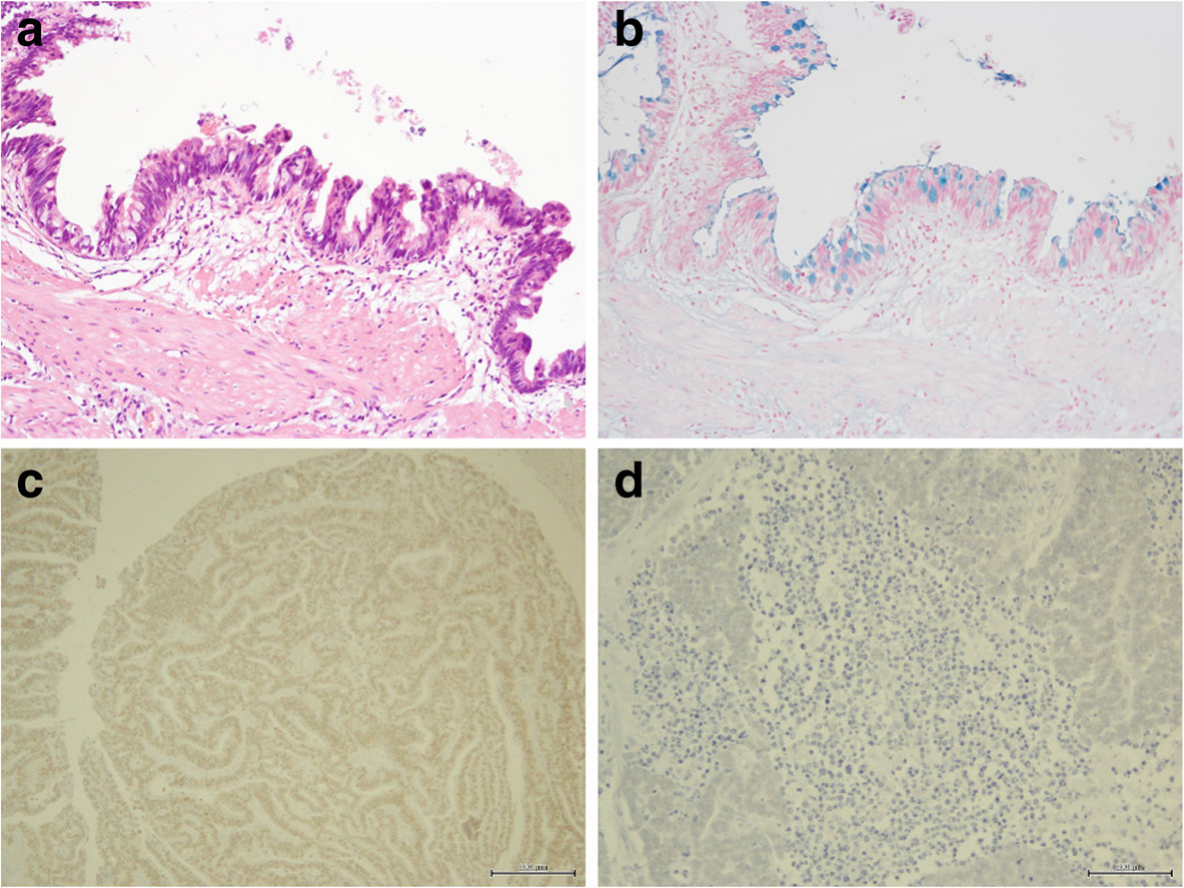
Staining for intestinal metaplasia. The epithelium around the tumor shows intestinal metaplasia with goblet cells (**a** H&E stain, ×100; **b** alcian blue stain, ×100). **c** The adenocarcinoma component and intestinal metaplastic epithelium in the gallbladder are positive for CDX2 immunohistochemical stain (×200), **d** but the neuroendocrine component is negative for CDX2 (×200)

As the result of thorough pathological re-evaluation by total segmentation, a negligible area of adenocarcinoma was detected in the lymph nodes (Fig. 3e, f). The adenocarcinoma component and the intestinal metaplastic epithelium in the gallbladder were both positive for CDX2 (Fig. 4a), but the neuroendocrine component in hilar lymph nodes was negative for CDX2 (Fig. 4b).

Postoperatively, the patient received three cycles of carboplatin (area under the curve of 5 on day 1 repeated every 21 days) and etoposide (80 mg/m^2^ on days 1 through 3 repeated every 21 days). During the first course, grade 4 neutropenia occurred and it was managed with prophylactic fluoroquinolones. After 4 months, multiple recurrences in the para-aortic lymph nodes were detected, which was pathologically demonstrated via EUS-FNA to be NEC. The patient underwent second-line chemotherapy with amrubicin (24 mg/m^2^ on days 1 through 3 repeated every 21 days). Grade 4 neutropenia and anemia developed during the courses, and she needed to receive pegylated granulocyte colony-stimulating factor and red blood cell transfusion. However, she died of progressive disease 8 months after surgery.

### Discussion

We present a rare case of a gallbladder cancer with regional bulky lymph nodes composed histologically of NEC, which was different from the histology of the primary gallbladder adenocarcinoma. The neuroendocrine cells in the regional lymph nodes are reasonably concluded to originate from the gallbladder, taking into account the preoperative PET/CT findings as well as the postoperative pathological result that hilar lymph nodes contained a small amount of adenocarcinoma cells.

According to the 2010 WHO classification, mixed adeno-neuroendocrine carcinoma (MANEC) is defined as a tumor where both components exceed 30% of the neoplasm [3]. The clinicopathological features of biliary MANECs include the adenocarcinoma component being predominantly located at the surface of the tumor and areas with the majority of the invasive cells, such as lymph node metastases, often involving neuroendocrine components [4]. Therefore, our case does not fulfill the criteria for MANEC because of the tiny area of adenocarcinoma, and moreover, there have been no previous reports of this kind of pathological feature.

On the other hand, the exact histogenetic origin of gallbladder NETs remains uncertain because normal gallbladder mucosa does not contain neuroendocrine cells [5]. However, several hypotheses have been proposed [6, 7]. Chronic inflammation mostly due to cholelithiasis and subsequent intestinal metaplasia of the gallbladder has been assumed to be a precursor of gallbladder epithelial tumors [8, 9], and this theory, called the metaplasia–dysplasia–carcinoma cascade [10], is also suggested to be associated with neuroendocrine tumors [11–15]. CDX2, a homeobox gene, is involved in the differentiation and maintenance of enteric epithelium [16] and is known to be a useful immunohistochemical marker of intestinal metaplasia of the gallbladder [17]. Acosta et al. reported a case of gallbladder MANEC that was positive for CDX2 in both tumor components and concluded that the finding supported the metaplasia–dysplasia–carcinoma sequence [18]. In the present case which is non-matching as MANEC, the adenocarcinoma component of the primary tumor and the intestinal metaplasia lesions were positive for CDX2 but the neuroendocrine cells were not (Fig. 4c, d). Therefore, these findings may indicate that some mechanism other than a metaplastic change may have been involved in the oncogenesis of NEC in this case. Further analysis of oncogenic mutation may prove the relationship between gallbladder and lymph nodes. Since the neuroendocrine cells existed only in the hilar lymph nodes, which contained a small amount of adenocarcinoma, it may be plausible that the neuroendocrine cells in the lymph nodes arose from aberrant transformation of adenocarcinoma cells with slight neuroendocrine differentiation from the gallbladder as similar theories have been advocated by other authors [6, 19].

In the NCCN guidelines for resectable poorly differentiated or small cell tumors, surgical resection and chemotherapy with or without radiotherapy are advised, although definitive chemotherapy can be considered [20]. A recent study showed that patients with a Ki-67 index <55% were less responsive to platinum-based chemotherapy but had a longer survival than patients with higher Ki-67 indexes, and the authors concluded that patients with advanced gastrointestinal NEC with a Ki-67 index above 55% should be considered for chemotherapy without delay [21]. However, high-grade NEC originating from the gallbladder generally has a poor prognosis [1, 22, 23]. In our case, finally, through the Cancer Board discussion, we selected “carboplatin and etoposide” as first line chemotherapy. The primary treatment for high-grade NEC represents on the standard regimens employed for the treatment of small cell lung cancer, which are also recommended in NCCN guideline. “Amrubicin” was chosen as second line, based on previous reports [24–26]. Furthermore, since the cancer was confined to the gallbladder and regional lymph nodes in the present case, we proceeded to surgical resection before chemotherapy to prevent mechanical obstructive jaundice or cholangitis caused by bulky lymph nodes around the hepatic hilum. However, the tumor recurred 4 months after the surgery in spite of general chemotherapy. Most of extrapulmonary large or small cell carcinomas are aggressive and require combined multimodality treatment, and it should be planned for each patient based on the clinicopathological findings.

## Conclusions

We report a rare case of NEC of the hepatic hilar lymph nodes that were concomitant with an adenocarcinoma of the gallbladder. Based on the histopathological features of this case, some mechanism other than intestinal metaplasia may be involved in the oncogenesis of NEC arising from the gallbladder. High-grade NEC generally has a poor prognosis and planning an optimal therapeutic strategy for each patient is essential.

## Abbreviations

CA19-9: Carbohydrate antigen 19-9
CEA: Carcinoembryonic antigen
CT: Computed tomography
ENGBD: Endoscopic naso-gallbladder drainage
EUS-FNA: Endoscopic ultrasound-guided fine-needle aspiration
FDG: ^18^F fluorodeoxyglucose
H&E: Hematoxylin and eosin
MANEC: Mixed adenoneuroendocrine carcinoma
MRCP: Magnetic resonance cholangiopancreatography
NCCN: National Comprehensive Cancer Network
NEC: Neuroendocrine carcinoma
NET: Neuroendocrine tumor
NSE: Neuron-specific enolase
PET/CT: Positron emission tomography/computed tomography
WHO: World Health Organization
